# Analysis of impulse oscillometric measures of lung function and respiratory system model parameters in small airway-impaired and healthy children over a 2-year period

**DOI:** 10.1186/1475-925X-10-21

**Published:** 2011-03-25

**Authors:** Erika G Meraz, Homer Nazeran, Carlos D Ramos, Pat Nava, Bill Diong, Michael D Goldman

**Affiliations:** 1 Department of Electrical and Computer Engineering, The University of Texas at El Paso, El Paso, Texas, USA; 2 Universidad Autónoma de Ciudad Juárez, Chihuahua, México; 3 Department of Engineering, Texas Christian University, Fort Worth, Texas, USA; 4 Geffen School of Medicine, University of California at Los Angeles, California, USA

## Abstract

**Background:**

Is Impulse Oscillometry System (IOS) a valuable tool to measure respiratory system function in Children?

Asthma (A) is the most prevalent chronic respiratory disease in children. Therefore, early and accurate assessment of respiratory function is of tremendous clinical interest in diagnosis, monitoring and treatment of respiratory conditions in this subpopulation.

IOS has been successfully used to measure lung function in children with a high degree of sensitivity and specificity to small airway impairments (SAI) and asthma. IOS measures of airway function and equivalent electrical circuit models of the human respiratory system have been developed to quantify the severity of these conditions. Previously, we have evaluated several known respiratory models based on the Mead's model and more *parsimonious versions *based on fitting IOS data known as extended RIC (eRIC) and augmented RIC (aRIC) models have emerged, which offer advantages over earlier models.

**Methods:**

IOS data from twenty-six children were collected and compared during pre-bronchodilation (pre-B) and post- bronchodilation (post-B) conditions over a period of 2 years.

**Results and Discussion:**

Are the IOS and model parameters capable of differentiating between healthy children and children with respiratory system distress?

Children were classified into two main categories: Healthy (H) and Small Airway-Impaired (SAI). The IOS measures and respiratory model parameters analyzed differed consistently between H and SAI children. SAI children showed smaller trend of "growth" and larger trend of bronchodilator responses than H children.

The two model parameters: peripheral compliance (Cp) and peripheral resistance (Rp) tracked IOS indices of small airway function well. Cp was a more sensitive index than Rp. Both eRIC and aRIC Cps and the IOS Reactance Area, AX, (also known as the "Goldman Triangle") showed good correlations.

**Conclusions:**

What are the most useful IOS and model parameters?

In this work we demonstrate that IOS parameters such as resistance at 5 Hz (R5), frequency-dependence of resistance (fdR: R5-R20), reactance area (AX), and parameter estimates of respiratory system such as Cp and Rp provide sensitive indicators of lung function and have the capacity to differentiate between obstructed and non-obstructed airway conditions. They are also capable of demonstrating airway growth-related changes over a two-year period.

We conclude that the ***IOS parameters AX ***and the ***eRIC model derived parameter Cp ***are the most reliable parameters to track lung function in children before and after bronchodilator and over a time period (2 years).

Which model is more suitable for interpreting IOS data?

IOS data are equally well-modelled by eRIC and aRIC models, based on the close correlations of their corresponding parameters - excluding upper airway shunt compliance. The eRIC model is a more *parsimonious *and equally powerful model in capturing the differences in IOS indices between SAI and H children. Therefore, it may be considered a clinically-preferred model of lung function.

## Background

Asthma is an inflammatory condition of the airways resulting in airway hyperactivity and generating increased mucus, mucosal swelling and airway smooth muscle contraction, all of which contribute to (partial) airway obstruction. The symptoms include chest tightness, coughing and wheezing, and in severe cases shortness of breath and low blood oxygen [1]. According to Dorland's medical dictionary small airway impairment is a chronic obstructive bronchitis with narrowing of the bronchioles and small bronchi.

According to the American Academy of Allergy, Asthma and Immunology, asthma and allergies strike 1 out of 4 Americans and approximately 20 million Americans have asthma. Nine million children under 18 in the U.S. have been diagnosed with asthma. Every day in America 40,000 people miss school or work, 30,000 have an asthma attack, 5,000 visit the emergency room, and 1,000 are admitted to a hospital and, although asthma is rarely fatal, 11 persons die every day due to this condition. Direct health care costs for asthma in the U.S. total more than $10 billion annually; and indirect costs (lost productivity) are $8 billion resulting in a total of $18 billion [2].

In Mexico, 10% of the population (approximately 10 million people) suffer from asthma. It is the most common cause of chronic illnesses and emergency hospitalizations in children according to the Mexican College of Allergy, Asthma and Pediatric Pulmonology [3].

Assessment of respiratory function is important in diagnosis and monitoring of asthma and other respiratory diseases in children [4]. The pulmonary function test most commonly used to detect small airway impairment and asthma is spirometry, which measures the volume of air that can be moved in or out of the lungs as a function of time with rapid and maximal inspiratory and expiratory efforts. This requires a considerable degree of cooperation from the subject, which is difficult to achieve for older children and cannot be achieved by younger children. This makes the diagnosis of small airway impairment and asthma difficult owing to the lack of objective measurements for younger children [5]. Furthermore, it has been reported that some asthmatic patients do not improve spirometrically, despite clinical improvement with treatment [6]. This is of concern, because if asthma is not appropriately controlled, it can lead to permanent airway damage.

In contrast to forced spirometry, Forced Oscillation Technique (FOT) superimposes small air pressure perturbations on the natural breathing of a subject to measure the mechanical properties of the lungs. The Impulse Oscillometry System (IOS) uses this technique and measures respiratory impedance using short pulses (impulses) of air pressure. Impulse oscillometry has been developed as a patient-friendly lung function test that minimizes demands on the patient and requires only passive cooperation of the subject wearing a nose clip, keeping lips tightly closed about a mouthpiece and breathing normally through the mouth. IOS has been used with success to assess lung function in healthy and asthmatic children as well as adolescents [4-28].

IOS yields frequency-dependent curves of respiratory impedance that are visually analyzed to recognize changes in shape and magnitude of the curves and distinguish healthy respiratory function from its diseased state. IOS data can be deployed to develop mechanical and equivalent electrical circuit models of the respiratory impedance to evaluate and quantify lung mechanics. In these equivalent models, electrical components analogous to mechanical resistance, compliance, and inertance inherent in the respiratory system are used. Therefore, estimates for these model parameters based on IOS measurements could be used as baseline measures for better detection, diagnosis, and treatment of different respiratory diseases [29].

In infants and children reversible airway obstruction and bronchial hyper-responsiveness (BHR) are significant components contributing to the diagnosis of bronchial asthma [27]. According to a recently developed document on Pulmonary Function Testing in Preschool Children (2007), FOT has been successfully performed in different settings, and a number of studies have demonstrated that FOT was capable of identifying airway obstruction and reactions to bronchodilators and broncho-constrictors [30]. Several studies have been developed to assess bronchodilator responses using FOT. Marotta et al [7] performed a study in 4-year old children concluding that IOS bronchodilator responses are remarkably abnormal in this population (children presented a significant bronchodilator response), and that IOS is a useful diagnostic tool in detection of early asthma development. Oostveen et al [31] performed a comprehensive review on methodology, recommendations and future developments of FOT in clinical practice stating that FOT is a reliable method to assess bronchial hyper-responsiveness in adults and children. Ortiz et al [8] performed an IOS study in children 2 to 5 years old in El Paso, Texas, finding that IOS is an acceptable method of assessing airway responses to bronchoactive drugs in this age group. In a more recent study related to the use of FOT to detect bronchodilation in children, Bar-Yishay et al [32] concluded that FOT could reliably measure response to bronchodilator therapy. Recently Song et al [13] researched the utility of impulse oscillometry in young children with asthma finding that asthmatic children differed from control subjects in IOS-assessed bronchodilator response and that there were some significant correlations between bronchodilator responses of spirometric and IOS parameters. Galant et al [33] stated that bronchodilator response (BDR) would appear to give important additional information about airway inflammation and found that IOS is a promising test to identify asthmatic preschoolers. In the same way Jee et al [34] suggested that Xrs5 might be a useful parameter for IOS-assessed bronchial challenge testing in preschool children with asthma.

All this evidence confirms that lung function in children and adolescents is sensitively and accurately assessed by IOS, before and after bronchodilation. Nevertheless few longitudinal Forced Oscillation (FO) data exist in healthy subjects or in those with airflow obstruction. Oostveen et al [31] noted the need for a practical FO index to define airway obstruction.

Previous work by our research group has focused on development and analysis of different equivalent electrical circuit models of human respiratory impedance. Our efforts to date, have demonstrated that the performance of the extended RIC (eRIC) and augmented RIC (aRIC) models rank in the middle of a series of conventional models developed over the past several decades in terms of total cumulative error. However, they provide parameter estimates that are physiologically more realistic and in line with expected values in healthy subjects and those suffering from pulmonary diseases [29,35-42], than previous models.

The present study is proposed to determine sensitivity of IOS parameters to show growth-related changes (increases or decreases), over a two-year period, and to analyze eRIC and aRIC model parameter estimates of lung function in Healthy (H) and Small Airway Impaired (SAI) children, to evaluate the performance of these models in quantifying airway function in this population and to show the correlation of these model parameters with IOS measures. In this paper we use the terms Healthy (H) and Normal (N) interchangeably.

## Methods

### Subjects

For this investigation, twenty six children were recruited and tested. A total of 15 males and 11 females were included in this study. Children were first classified, by our expert clinician, as being part of one of four groups: Healthy (Normal), probable SAI (PSAI), SAI and Asthmatic, based on initial baseline IOS studies in 2006 using data of Clement et al [43] as a guide to normality. We requested subjects studied in 2006 to return in 2008 for more complete IOS testing pre- and post-bronchodilation. All twenty six subjects returned for re-evaluation in 2008.

In a previous study by our research group [44] it was observed that children classified as H or PSAI were relatively similar in both IOS and aRIC model parameters. In the same way it was observed that differences between SAI and asthmatic children were similarly modest. Therefore, it was concluded then that while expert clinician diagnostic classification distinguished between children based on 4 levels of perceived normality or absence thereof from the visual patterns of IOS data, group mean IOS and aRIC data appear to fall into two distinctly different groups: either healthy or small airway impaired. For this reason children in this research were classified as being part of one of two groups: H or SAI. Nineteen children were IOS classified as SAI, and seven as H. Age, height and weight Mean ± SD values and ranges for this population, in 2006, are presented in Table 1.

**Table 1:** Study Population in 2006.

Subject	Males and Females	
	
Demographics	Range	Mean ± SD
Age (years)	5 to 14	8.7 ± 2.8
Height (cm)	110.7 to 171.7	135.2 ± 20.4
Weight (kg)	19.1 to 72.7	36.1 ± 16.9

In their first tests, in 2006, children were tested without the use of a bronchodilator (B), and in their second tests in 2008 children were tested before and after using a B (pre-B and post-B). The bronchodilator used was levalbuterol. Parents or caregivers were asked to carefully read and sign a consent form, and children from 5 to 17 years were asked to sign an assent form, both forms approved by the University of Texas at El Paso IRB. Parents or caregivers were also asked to complete a questionnaire regarding child demographic and household information, child medical history (checklist for asthma, symptom frequency, nocturnal symptoms, triggers, medications, and health conditions), family medical history, smoking and environmental tobacco smoke exposure.

### IOS Testing

The IOS superimposes small air pressure perturbations on the natural breathing of a subject to measure the impedance of the respiratory system. The *respiratory Impedance *(Z) measured by IOS consists of *respiratory Resistance *(R) and *respiratory Reactance *(X) and includes hallmarks such as *Reactance Area *(AX) also known as the "***Goldman Triangle***" over a selected frequency range of 3 to 35 Hz. *Z *is the transfer function or ratio of the Fourier transform of the pulses of air pressure and the consequent air flow perturbations. It is a mathematical complex quantity with real and imaginary parts. The real part corresponds to the R, which includes the resistance of the proximal and distal airways (central and peripheral), as well as lung tissue and chest wall. Usually, central resistance is dominant, depending on airway calibre and the airway walls surface, while lung tissue and chest wall resistances are usually negligible. In healthy adult subjects, R is almost independent of oscillation frequency. When an airway obstruction occurs, either central or peripheral, R5 (Resistance at 5 Hz) is increased above normal values. Central airway obstruction elevates R evenly independent of oscillation frequency. Peripheral airways obstruction is highest at low oscillation frequencies and falls with increasing frequency; this is called the negative frequency-dependence of Resistance (fdR). As peripheral resistance increases, R becomes more frequency dependent. Small children normally present frequency-dependence of resistance and this may be greater than in adults in the presence of peripheral airflow obstruction [45]. The imaginary part of Z is the *respiratory Reactance *(X), which includes the mass-inertive forces of the moving air column expressed in terms of *inertance *(I) and the elastic properties (compliance) of lung periphery expressed in terms of *capacitance *(C) [45]. Resistance and Reactance are measured in cmH_2_O/L/s or KPa/L/s. The Resonant Frequency (*F*_*res*_) is the point at which reactance is zero and is measured in Hertz (1/s) [7]. The Reactance Area (AX - *"Goldman Triangle"*) is the integrated low frequency respiratory reactance magnitude between 5 Hz and *F*_*res*_, and it is measured in cmH_2_O/L or KPa/L. AX is a practical FO index related to respiratory compliance. AX is a single quantity that reflects changes in the degree of peripheral airway obstruction and closely correlates with fdR [45].

A Jaeger MasterScreen IOS (Viasys Healthcare, Inc. Yorba Linda, CA, USA) was used in this study. The system was calibrated every day using a 3-L syringe for volume calibrations and a reference resistance (0.2 KPa/L/s) for pressure calibrations. Children were asked to wear a nose clip, while breathing normally through a mouthpiece and were instructed to tightly close their lips around it to avoid air leakage. Three to 5 IOS test replicates were performed on each subject to ensure reproducible tests without artefacts caused by air leaks, swallowing, breath holding or vocalization [9]. In each IOS test impulses were applied for a period of 30 to 45 seconds. IOS data were carefully reviewed off line and quality-assured by our expert clinician to reject segments affected by airflow leak or swallowing artefacts.

### Respiratory Impedance Models

**Figure 1:**
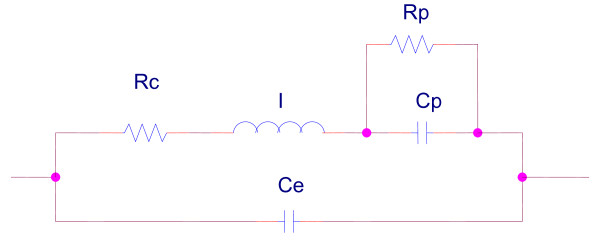
**Augmented RIC model **[14].

The aRIC respiratory model was proposed as an improvement to the eRIC model [38] and it can be considered as a simplification of the Mead's model [36] when both lung and chest wall compliances are very, very large. The aRIC model has the same components as the eRIC model, having only a additional compliance. aRIc is composed of central (large airway) Resistance (Rc), large airway Inertance (I), peripheral (small airway) Compliance (Cp), peripheral (small airway) Resistance (Rp) and an additional compliance Ce (see figure 1), representing extrathoracic Compliance, which accounts for any increase in the real part of the respiratory system's impedance at the higher frequencies due to upper airways shunt effects.

The parameters for the eRIC and aRIC (Rc, R_p_, I, C_p_, and C_e_) models were estimated using average resistance and reactance values of the IOS parameters at different frequencies (3, 5, 10, 15, 20, 25 Hz) for the 26 children. These parameter estimation tasks were carried out by using parameter estimation algorithms developed in Matlab.

### Data Analysis

The following IOS parameters: R_5 _(Resistance at 5Hz), R_5 _- R_20 _(an index of frequency-dependence of resistance) and Reactance Area, AX (an integrative index of low-frequency reactance), were tabulated to assess expected growth-related, and Bronchodilation-related (B-related) changes in R and X magnitudes. These IOS parameters have been reported to be sensitive measures for detecting changes in *bronchomotor tone *in adolescent asthmatic subjects [9]. R_5 _and R_5 _- R_20 _were used as sensitive indices of *peripheral airway obstruction*. R_5 _- R_20 _is believed to reflect small airway impairment, and it has also been reported that AX is sensitive to changes in the degree of peripheral *airflow obstruction *[9].

It has been previously demonstrated that one parameter of the aRIC model corresponding to peripheral lung compliance (Cp) provided good discrimination between asthmatic and normal children [36]. It has also been shown that Rp and Cp presented significant differences between pre- and post-bronchodilation conditions [46]. Therefore, these two model parameters (Rp and Cp) were also analyzed in this research. Statistical analyses were performed using paired Student's t-test and a p < 0.05 was considered as significant.

## Results

Table 2 shows average values of IOS parameters (R5, R5-R20, and AX) and statistical significance of differences between *Healthy (H), also called Normal (N)*, and SAI subjects at baseline in 2006 pre-B and at pre-B and post-B conditions in 2008. It is observed that all IOS parameters (R5, R5-R20 and AX) showed significant differences between SAI and the *H or N *group.

**Table 2:** Average Values and Statistical Significance (SAI vs H) of IOS parameters.

Tests	R5		p-value	R5-R20		p-value	AX		p-value
	(kPa/l/s)			(kPa/l/s)			(kPa/l/s)		
	**SAI**	**H**		**SAI**	**H**		**SAI**	**H**	
**2006 pre-B**	0.73	0.52	<0.002	0.31	0.15	<0.001	2.51	1.09	<0.0003
**2008 pre-B**	0.63	0.43	<0.001	0.27	0.13	<0.003	2.00	0.80	<0.002
**2008 post-B**	0.53	0.38	<0.02	0.20	0.08	<0.002	1.34	0.57	<0.01

Table 3 illustrates the statistical significance between *H *and SAI children in 2006 (baseline pre-B) and at pre-B and post-B conditions two years later for the eRIC and aRIC model parameters: Peripheral Resistance (Rp) and Peripheral Compliance (Cp). Rp failed to detect statistical significances between SAI and *H *groups, no significant differences were found between these two groups (p > 0.05, NS) in both models; while Cp showed significant differences between these two groups in both models.

**Table 3:** Average values and Statistical Significance (SAI vs H) of the eRIC and aRIC Model Parameters.

Tests	aRIC Rp		p-value	aRIC Cp		p-value	eRIC Rp		p-value	eRIC Cp		p-value
	(kPa/l/s)			(l^2/kPa^2s)			(kPa/l/s)			(l^2/kPa^2s)		
	**SAI**	**H**		**SAI**	**H**		**SAI**	**H**		**SAI**	**H**	
**2006 pre-B**	0.592	0.451	>0.05	0.053	0.100	**<0.02**	0.824	0.501	**<0.03**	0.045	0.115	**<0.0002**
**2008 pre-B**	0.470	0.438	>0.05	0.056	0.136	**<0.003**	0.601	0.472	>0.05	0.056	0.155	**<0.0002**
**2008 post-B**	0.359	0.316	>0.05	0.067	0.162	**<0.001**	0.472	0.386	>0.05	0.076	0.173	**<0.0001**

Table 4 demonstrates significant differences between 2006 pre-B and 2008 pre-B, and between 2008 pre-B and post-B data for IOS parameters. R5, R5-R20 and AX showed significant differences for SAI group comparing both scenarios (2006 pre-B vs 2008 pre-B; and 2008 pre-B vs 2008 post-B). In the *H or N *group R5, R5-R20 and AX showed no significant differences (p > 0.05) in both scenarios with the exception of 2006 pre-B and 2008 pre-B for R5.

**Table 4:** Significance difference between 2006-2008 pre-B, and 2008 pre-B and post-B data for IOS parameters.

Tests	R5		R5-R20		AX	
	(kPa/l/s)		(kPa/l/s)		(kPa/l/s)	
	p-value		p-value		p-value	
	**SAI**	**H**	**SAI**	**H**	**SAI**	**H**
**2006-2008 pre-B**	**<0.0002**	**<0.02**	**<0.05**	>0.05	**<0.0005**	>0.05
**2008 pre-B/post-B**	**<0.0001**	>0.05	**<0.002**	>0.05	**<0.00005**	>0.05

Table 5 demonstrates significant differences between 2006 pre-B and 2008 pre-B, and between 2008 pre-B and post-B data for model parameters. For *H or N *children both models parameters, Rp and Cp, presented no significant differences (p > 0.05) between 2006-2008 pre-B conditions and 2008 pre-B and post-B data. In SAI children both model parameters, Rp and Cp, showed significant differences between 2006-2008 pre-B situations and 2008 pre-B and post-B data, with the exception of aRIC Cp, which showed no significant differences (p > 0.05) between 2006-2008 pre-B data.

**Table 5:** Significant differences between 2006-2008 pre-B, and 2008 pre-B and post-B data for model parameters.

Tests	aRIC Rp		aRIC Cp		eRIC Rp		eRIC Cp	
	(kPa/l/s)		(l^2/kPa^2s)		(kPa/l/s)		(l^2/kPa^2s)	
	p-value		p-value		p-value		p-value	
	**SAI**	**H**	**SAI**	**H**	**SAI**	**H**	**SAI**	**H**
**2006-2008 pre-B**	**<0.002**	>0.05	>0.05	>0.05	**<0.002**	>0.05	**<0.01**	>0.05
**2008 pre-B/post-B**	**<0.000002**	>0.05	**<0.05**	>0.05	**<0.003**	>0.05	**<0.02**	>0.05

In Table 6 we can observe the growth and bronchodilation percentage of change for *H or N *and SAI children from 2006 pre-B to 2008 pre-B, and from pre-B and post-B in 2008. A negative sign represents a decrease in magnitude, and a positive sign represents an increase.

**Table 6:** Growth and bronchodilator percentage of change in H and SAI children.

IOS and	2006 pre-B - 2008 preB	2008 preB - 2008 postB	2006 pre-B - 2008 preB	2008 preB - 2008 postB
Model	% of change for	% of change for	% of change for	% of change for
Parameters	Healthy	Healthy	SAI	SAI
R5	-17	-12	-14	-16
R5-R20	-13	-38	-13	-26
AX	-27	-29	-20	-33
aRIC Rp	-3	-28	-21	-24
aRIC Cp	35	19	4	20
eRIC Rp	-6	-18	-27	-21
eRIC Cp	35	12	25	35

From Tables 4, 5 and 6 we can make the following observations:

*In **children with SAI**, the three IOS parameters analyzed decreased from 2006 pre-B to 2008 pre-B: R5 (14%, p < 0.0002), R5-R20 (13%, p < 0.05), and AX (20%, p < 0.0005). Comparing 2008 pre-B and post-B data, post-B decreases in all IOS parameters were larger (16-33%, p < 0.002). Within the eRIC and aRIC model parameters eRIC Rp had a higher decrease from 2006 pre-B to 2008 pre-B (27%, p < 0.002) than aRIC Rp (21%, p < 0.002); similarly, eRIC Cp had a significantly higher increase (25%, p < 0.01) than aRIC Cp (4%), showing no significant differences (p > 0.05) between groups. Comparing 2008 pre-B and post-B data, eRIC Rp had a decrease (21%, p < 0.003) and aRIC Rp had a slightly higher decrease (24%, p < 0.000002), while eRIC Cp had a higher increase (35%, p < 0.02) than aRIC Cp (20%, p < 0.05).

*For **children without SAI (***H or N***)**, growth-related R and X magnitudes decreased from 2006 pre-B to 2008 pre-B (13-27%, p < 0.02 for R5 only), R5-R20 and AX showed no significant differences (p > 0.05); post-B decreases were larger (12-38%), and all IOS parameters showed no significant differences. In this group of children, the eRIC and aRIC model parameters presented the following changes from 2006 pre-B to 2008 pre-B: eRIC Rp decreased (6%, p > 0.05) more than aRIC Rp (3%, p > 0.05) both with no significant differences; while eRIC Cp and aRIC Cp showed equal increases and no significant differences (35%, p > 0.05). Comparing 2008 pre-B and post-B eRIC Rp had a smaller decrease (18%, p > 0.05) than aRIC Rp (28%, p > 0.05) both showing no significant differences; eRIC Cp had also a smaller increase (12%, p > 0.05) than aRIC Cp (19%, p > 0.05) and also no significant differences.

It was observed that in 2006 one child was overweight, he was classified into the group of SAI subjects, and it was interesting to analyze the impact of including him or not in the calculation of the R and X results for 2006. In table 7 the impact of including or excluding the overweight subject on calculation of the R and X averaged values in 2006 can be observed.

**Table 7:** Impact on calculation of R and X (averaged values) by including or excluding an overweight child in 2006 (OC = Overweight child included and NOC = No overweight child included).

Frequency	Resistance (kPa/l/s)		Reactance (kPa/l/s)	
(Hz)	2006	SAI	2006	SAI
	OC	NOC	OC	NOC
3	0.86	0.86	-0.40	-0.41
5	0.72	0.71	-0.32	-0.33
10	0.55	0.54	-0.21	-0.21
15	0.43	0.42	-0.12	-0.12
20	0.41	0.40	0.02	0.02
25	0.47	0.46	0.11	0.12
35	0.59	0.59	0.20	0.21

The table shows that the average values for R and X are very similar. Therefore, including a child with overweight did not have a considerable impact on the results presented in this research.

**Figure 2:**
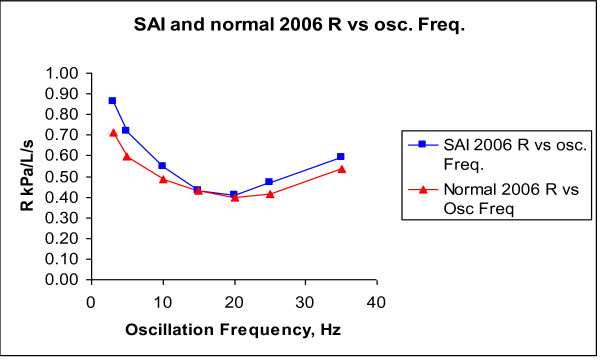
**R vs Oscillation Frequency in 2006 for averaged SAI and averaged H subjects**.

**Figure 3:**
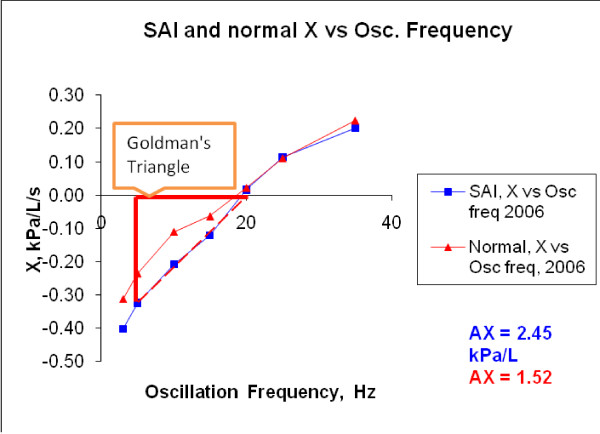
**X vs Oscillation Frequency in 2006 for averaged SAI and averaged H subjects**.

In figures 2 and 3, IOS Resistance (R) and Reactance (X) are shown as a function of different oscillation frequencies in 2006 (baseline) for averaged *H or N *and averaged SAI children.

Figure 3 exemplifies the values of AX, showing that it is larger, as expected, for children with SAI (AX can be visualized as the triangular area from X5 to Fres: the point at which X = 0).

**Figure 4:**
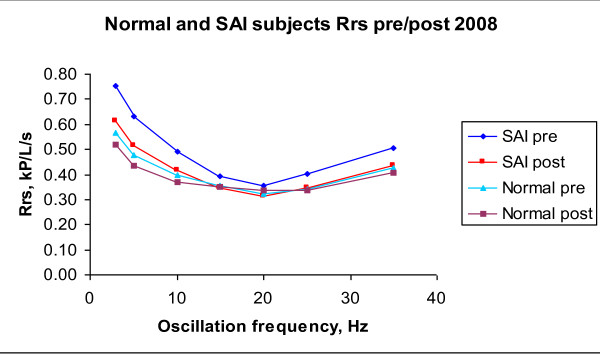
**R vs Oscillation Frequency in 2008 for averaged SAI and averaged H subjects**.

**Figure 5:**
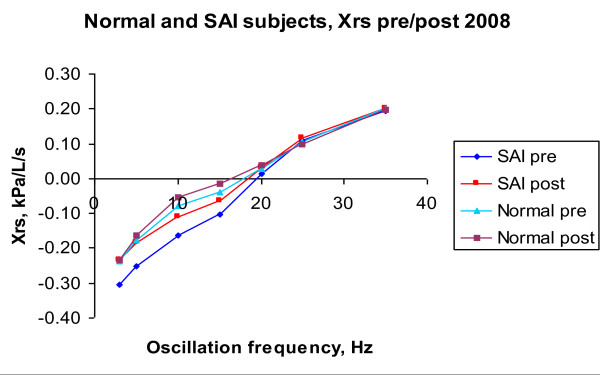
**X vs. Oscillation Frequency in 2008 for averaged SAI and averaged H subjects**.

Figures 4 and 5 illustrate 2008 IOS data for R (Rrs) and X (Xrs) vs. oscillation frequency under pre-B and post-B conditions, for averaged *H *and averaged SAI children. It can be observed, in both graphs, that H pre-B line is close to SAI post-B line.

**Figure 6:**
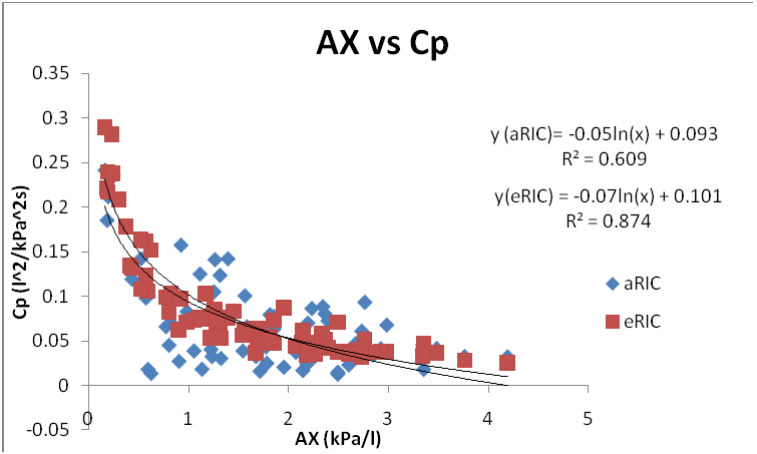
**Cp as a function of AX for the eRIC and aRIC models in all subjects and measurements (2006 pre-B, 2008 pre-B and 2008 post-B)**.

In figure 6 we can observe the regressions between AX and Cp for both models (eRIC and aRIC) in all subjects and measurements (2006 pre-B, 2008 pre-B, and 2008 post-B). The regression for Cp as a function of AX is very similar in both models, but a better correlation was found for the eRIC Cp (r = 0.935) than for the aRIC Cp (r = 0.780).

**Figure 7:**
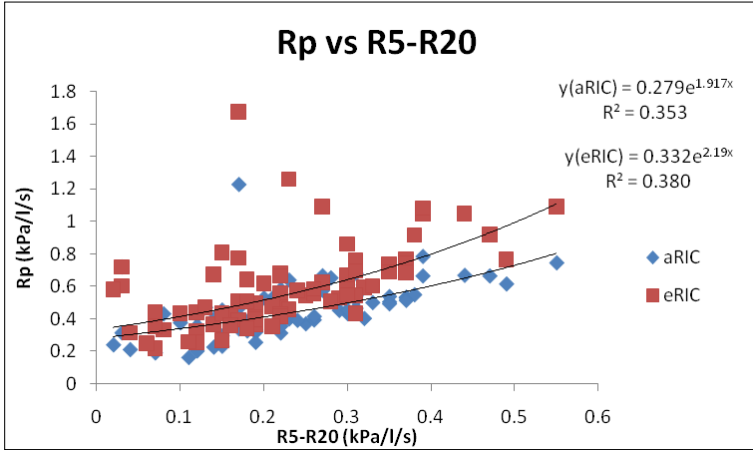
**Rp as a function of R5--R20 for the eRIC and aRIC models in all subjects and measurements (2006 pre-B, 2008 pre-B and 2008 post-B)**.

Figure 7 shows the regressions for both models (eRIC and aRIC) Rp and R5-R20 in all subjects and measurements (2006 pre-B, 2008 pre-B and 2008 post-B), where both produced smaller correlations than the previous correlations presented in figure 6 (AX vs Cp). We can observe that both model Rp regressions vs. R5-R20 are almost the same (eRIC r = 0.616 and aRIC r = 0.594).

**Figure 8:**
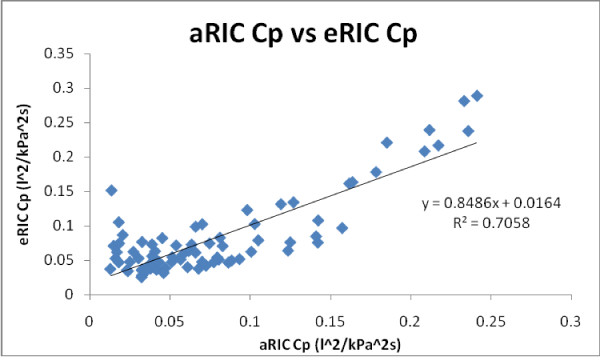
**Regression line for both models (aRIC and eRIC) Cp in all subjects and measurements (2006 pre-B, 2008 pre-B and 2008 post-B)**.

Figure 8 illustrates aRIC Cp values plotted as a function of eRIC Cp. Both models' parameters presented a close correspondence (slope = 0.8486 and r = 0.840).

An almost equal correlation was found between aRIC Rp and eRIC Rp (slope = 1.223 and r = 0.830). Lower regression values were found for central (large airway) resistance Rc and large airway inertance I, Rc's slope was 0.457 and r = 0.544, meanwhile for Inertance, the slope was 0.208 and r = 0.480. These results demonstrate very similar parameter estimates for Cp and Rp from both models, and comparable estimates for Rc and I from both of them as well.

**Figure 9:**
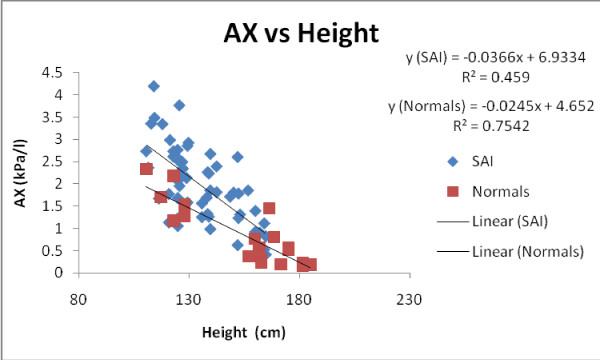
**AX vs Height in all subjects and measurements (2006 pre-B, 2008 pre-B and 2008 post-B)**.

**Figure 10:**
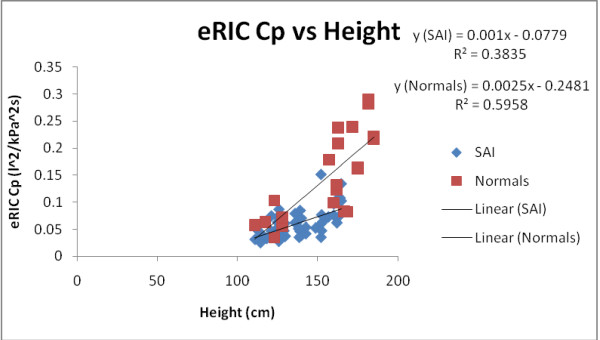
**eRIC Cp vs Height in all subjects and measurements (2006 pre-B, 2008 pre-B and 2008 post-B)**.

Figure 9 shows AX vs Height in all subjects and measurements (2006 pre-B, 2008 pre-B and 2008 post-B), while figure 10 depicts eRIC Cp vs Height in all subjects and measurements (2006 pre-B, 2008 pre-B and 2008 post-B). As it can be observed from both graphs, AX and Cp provide good discrimination between the SAI and *H or N *children. We can observe in figure 9 that *H *children have smaller values of AX as mentioned before and as expected. In figure 10 we can observe that *H *(Normals) children have higher values of Cp than SAI children, suggesting that SAI subjects present reduced respiratory compliance due to small airway inflammation and lumen diameter reduction. The overlapping points between SAI and *H *children trend lines in both graphs for Cp and AX (points where the trend lines are close together) could be explained by the previous observation made about pre-B data in *H *children being similar to post-B data in SAI children.

In table 8 we can observe the time change in R5, AX and eRIC Cp averaged data for SAI and *H *children. It can be observed that values of R and AX decreased with time (from 2006 pre-B to 2008 pre-B) and also showed a decrease with bronchodilation (2008 pre-B and 2008 post-B data) as expected; noticing that the largest decrease occurs in AX and is observed in pre- and post-B data for SAI children suggesting that AX can be a better discriminating parameter than R5 between SAI and *H *children.

**Table 8:** Time change in SAI and H children IOS and model parameters (averaged values).

Tests	SAI			Healthy		
	
	R5	AX	eric Cp	R5	AX	eric Cp
	(kPa/l/s)	(kPa/l/s)	(l^2/kPa^2s)	(kPa/l/s)	(kPa/l/s)	(l^2/kPa^2s)
**2006 pre-B**	0.73	2.51	0.045	0.52	1.09	0.115
**2008 pre-B**	0.63	2.00	0.056	0.43	0.80	0.155
**2008 post-B**	0.53	1.34	0.076	0.38	0.57	0.173

For the eRIC Cp parameter we can see an increase with time (from 2006 pre-B to 2008 pre-B) and also with bronchodilation (2008 pre-B and 2008 post-B data) also as expected. We can observe the largest increase in *H *children with time change (from 2006 pre-B to 2008 pre-B) meaning that *H *children's peripheral compliance increases with growth better than with bronchodilation and to a better extent than for children with SAI.

Zeltner et al. [47] performed a study about postnatal development and growth of the human lung, concluding that this process is made of three overlapping stages: (a) the *alveolar formation stage*, which begins in the final stage of the fetal life (36^th ^week) and ends between 1 and 1.5 years post partum, (b) a *stage of microvascular maturation*, thought to extend from the first month after birth to the age of 2 to 3 years, (c) the *normal growth period *starts after the microvascular maturation stage and lasts until body growth stops, during this period lung development is considered complete, then normal growth comprises only normal increase in lung size. Then it merges into a period of stable lung dimensions, until aging sets in. This study confirms a previous study about postnatal human lung growth [48] where it is stated that there is rapid alveolar multiplication during the first two years of life, and there is little or no increase in the total number of alveoli after the age of 2 years. It was also stated by Zeman et al. [49] in a more recent study about small airways and alveoli that from childhood (age 6 years) to adulthood, the number of respiratory units is maintained constant, while both the smallest bronchioles and alveoli increase in size to produce the enlarged lung volume with increased age and height. In healthy children growth resistance of the lungs is expected to decrease with age.

Mild to moderate asthma results in a pattern of airway obstruction that increases in magnitude from age 5 to 18 years [50]. Several studies have shown that asthma results in a reduced acceleration of lung growth [51]. Lung function in children with severe asthma is reduced in childhood years and decline in adult life to levels consistent with adult obstructive lung disease. This is the reason why early detection and treatment to prevent airway remodelling in childhood is extremely important as it may reduce the risk of long term complications of childhood asthma [52].

Elastic recoil (compliance) of the lungs is low in young children and increases with age; therefore it is possible that asthma could result in a failure for this increase in elastic recoil development [51]. For this reason in this research it was decided to analyze Cp (peripheral Compliance) as a measure of lung periphery elastic properties in the two years period.

**Figure 11:**
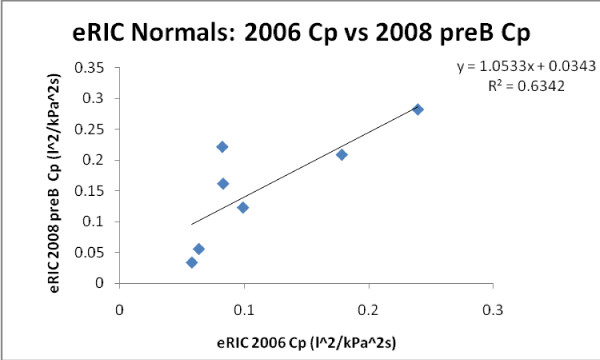
**Growth changes in eRIC Cp for all H children**.

**Figure 12:**
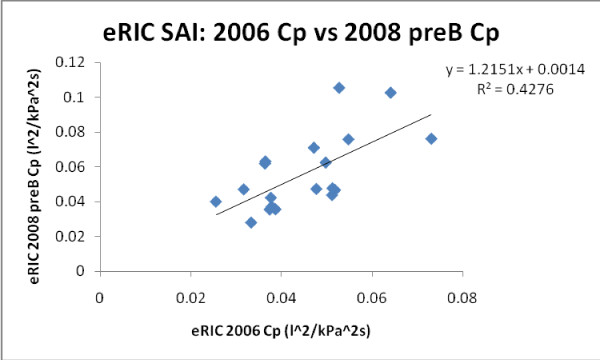
**Growth changes in eRIC Cp for all SAI children**.

Figures 11 and 12 exemplify growth related changes in eRIC Cp in all *H *and SAI children. A better correlation was found for eRIC Cp *H *children (slope = 1.053 and r = 0.796), comparing 2006 pre-B vs 2008 pre-B data, than for SAI children (slope = 1.2151 and r = 0.654).

A similar but slightly better correlation was found for aRIC Cp in *H *children (slope = 0.877 and r = 0.70), comparing 2006 pre-B vs 2008 pre-B data, than for SAI children (slope = 0.562 and r = 0.426).

Figure 11 illustrates growth development in Healthy children, and figure 12 could imply a reduced acceleration of lung growth in children with SAI.

## Discussion

For a long time, small airways have been considered to be the area of focus in asthma and there is already a substantial body of evidence to support the importance of small airways disease in asthmatic patients [53,54]. Small (peripheral) airways refer to about 7 to 19^th ^generation airways with an inner diameter of about 2 to 0.5 mm [55-57]. These airways are considered to be an important site of inflammation in both early chronic obstructive pulmonary disease and asthma. It is estimated that small airways resistance contributes 15 to 24% of airway resistance in healthy (normal) people and much higher in patients with severe diseases. It has also been stated that for subjects with nonasthmatic allergic disease (atopy) early manifestation prior to asthma could be early Small Airway Disease (SAD) also known as Small Airway Impairment and then if inflammation persists, asthma would appear [55].

Even though the importance of the small airways in asthma and other pulmonary diseases is established, clinical assessment of the peripheral airways continues to be a challenge to date [57] and a means of studying the small airways have never been readily available to clinicians [54]. The silent zone of the lung (small airways) can became a seat of inflammation and fibrosis from varied sources like Small Airway Impairment leading to small airways distortion ending in functional abnormalities. These abnormalities are evident because of the increased resistance to airflow at a lately detected stage when the condition has become severe. Such circumstances demand early diagnosis to prevent pulmonary complications [58].

Asthmatic patients present a progressive deterioration of lung function, and this deterioration seems to be more evident in younger asthmatics whose disease is not well controlled. Therefore, early evaluation and therapy for small airways might be even more effective when started earlier in the course of the disease [54].

It is observed that in healthy lung growth, airway resistance decreases with age. Peripheral resistance increases significantly in asthmatics with airflow obstruction compared with central resistance, suggesting that the peripheral airways are the predominant site of airflow obstruction in asthma [57].

An effective means to evaluate small airways performance could be achieved by integrating realistic models of lung function based on physiological measurements made by FOT and other techniques [53]. In this research we aimed to analyze and offer sensitive measures for healthy and impaired respiratory conditions such as SAI, by using the well-established IOS parameters R5, R5-R20 and AX, as well as the aRIC and eRIC model parameters such as Cp and Rp.

The significant differences found between SAI and *H *groups, illustrated in tables 2 and 3, confirm the ability of the analyzed IOS parameters: R5, R5-R20 (fdR) and AX, as well as model parameter Cp (for both aRIC and eRIC models) to differentiate between obstructed and non-obstructed airways. It is also demonstrated in these tables that values of IOS parameters and both Rps in both models are higher in children with SAI than in *H *children. It is also clear that Cps for both models are higher in *H *children than in children with SAI, as expected.

In tables 4 and 5, the significant differences observed in the SAI group in all IOS parameters (R5, R5-R20 and AX) and model parameters (eRIC and aRIC Rp, and eRIC Cp), comparing baseline (pre-B) data in 2006 with pre-B 2008 data suggest an abnormal lung growth development due to the presence of the illness in the two year period. In contrast no significant differences were seen for *H *children in the same parameters during the same period of time, with the exception of R5, which may be attributed to the noise previously reported to occur in low frequency resistance and reactance measurements [59].

As illustrated in figure 3, the AX parameter is greater in children with SAI than in *H *children. An improvement in lung function will imply a decrease in AX value and AX, as stated before, reflects small airway function [59]. In table 6 it can be observed that the AX decrease presented as percentage of change in baseline (pre-B 2006 and pre-B 2008) IOS data, is greater in *H *children than in children with SAI, a -27% for *H *children and a -20% for children with SAI (a negative sign means a decrement). These results imply that AX demonstrated a higher decrease in the two years period in *H *children, indicating improved lung function that could be attributed to normal lung growth in this group. Furthermore, AX showed a lower decrease in children with SAI suggesting an impaired lung growth due to their illness. In addition, as stated before, significant differences were observed in the SAI group comparing 2006 and 2008 baseline data and no significant (p > 0.05) differences were found in the *H *group in the same period. These results suggest that AX might be a good IOS index used to differentiate changes over time (2 years) in lung function (impaired and non-impaired).

These findings agree with a recent study performed by Larsen, et al. [26] comparing IOS AX parameter with spirometry parameters, where it was concluded that the pattern of improvement seen in AX (XA), over the course of therapy, suggest this test might detect alterations in airway mechanics not reflected by spirometry.

In a study on clinical applications of FOT, Goldman developed an integrated response index for X, AX [59], now called *the ****"Goldman Triangle"***, previously explained in this study. Goldman also explained the history of the phenomenon called "frequency-dependence of resistance (fdR)" in this study.

There are several studies which are in agreement with the results presented here about the IOS parameters fdR (R5-R20) and AX being the indices most closely related to small airway function [26,45,60-65]. Even though these research studies suggest the potential effectiveness of IOS parameters there are still concerns about the effects of upper airway structures (like upper airway shunt) [33,64]; and additionally there is a necessity for establishing normal values as well as reproducibility studies for IOS parameters [33].

Infant's airways structure and proportions are different than those of the adult, and the relative greater lung compliance may accentuate the functional differences [66]. In children with SAI, lung compliance is lower than that in normal or healthy children. As it is observed in table 3 for both models, the Cp is higher in *H *than in SAI children. An improvement in lung function will produce an increment in Cp value. In table 6 it can be observed that the eRIC Cp increment presented as percentage of change in baseline (2006-2008) IOS data, is greater in *H *children than in children with SAI, a 35% for *H *children and a 25% for children with SAI. This higher percentage of improvement in *H *children for Cp may indicate (as AX would), an improved lung function in *H *children (normal lung growth). Whereas a lower increment in SAI group could represent impaired lung function and growth. Similarly significant differences were observed for SAI group in eRIC Cp parameter comparing 2006 and 2008 baseline data. These results also suggest that the eRIC Cp may be a good index capable of differentiating changes over time (2 years) in lung function (impaired and non-impaired).

Goldman et al. [64] developed a similar study closely related to this research, in adolescents and young adults with Cystic Fibrosis, and in asthmatic adults, obtaining very similar results to our results previously reported, and stating that the eRIC model parameters are reliable and present a slightly better correlation with IOS parameters compared to the aRIC model parameters, concluding that the less complex and more intuitive eRIC model may be more suitable for clinical diagnosis and evaluation after treatment. Goldman et al. concluded that IOS indices of SAI are modelled similarly well with and without upper airway shunt capacitance (Ce) for good quality IOS data, and do not seem to be dependent on upper airway shunt capacitance. This is to be expected since the IOS indices are based on low frequencies up to 20 Hz, whereas the upper airway shunt capacitance in the aRIC manifests significant, increasing effects on respiratory impedance only at higher frequencies (above the resonant frequency).

## Conclusions

IOS parameters differed consistently between *H *and SAI children over a two-year period. SAI children showed smaller trend of "growth" in all IOS parameters (R5, R5-R20 and AX) comparing 2006 pre-B and 2008 pre-B data; and larger trend of bronchodilator responses than *H *children in R5, AX, eRIC Rp and Cp, as well as aRIC Cp parameters. *The AX and eRIC Cp parameters showed larger differences between pre-B and post-B data*.

The eRIC and aRIC model parameters Cp and Rp track IOS indices of small airway function. Peripheral airway compliance (Cp) is a more sensitive index than peripheral airway resistance (Rp). eRIC and aRIC Cp are significantly larger in *H *or normal than SAI children, showing larger p values for eRIC Cp; while for both models, Rp did not show significant differences between *H *and SAI children.

Model calculated parameters Rp and Cp are narrowly comparable between both analyzed models (aRIC and eRIC). In the same manner Rc and I similarly present a good correlation in both models.

Both eRIC and aRIC Cp parameters showed significantly good correlations with AX; with eRIC model resulting in a higher r value than aRIC model (eRIC r = 0.935 and aRIC r = 0.780).

In this research study in children with and without SAI (*Healthy*), the eRIC model parameters showed to be consistent and to some extent more closely correlated with IOS measures compared to the aRIC model parameters. As eRIC is more intuitive, less complex and a more parsimonious model, it may be considered a more suitable diagnostic tool for clinical applications than the aRIC model.

IOS lung function data are similarly well-modelled by the eRIC (without upper airway shunt compliance) and aRIC models (with upper airway shunt compliance), which are reduced versions of the popular Mead's model developed at Harvard several decades ago, based on the close correlations of their corresponding parameters excluding Ce. The eRIC model is a more parsimonious and equally powerful model in capturing the differences between SAI and *H *children, therefore it is presented as a clinically-preferred model of lung function based on IOS data.

In summary, we conclude that the ***IOS parameters AX ***and the ***eRIC model derived parameter Cp ***are the most reliable parameters to track lung function in children before and after bronchodilator and over a time period (2 years). *AX *(*the "Goldman Triangle"*), representing the integrated low frequency respiratory reactance magnitude between 5 Hz and *F*_*res *_, and the eRIC Cp corresponding to the peripheral (small airway) Compliance demonstrated superior diagnostic discrimination compared to all other parameters analyzed and emerged as useful and reliable indices of lung function in children.

Further work in a larger number of *H *and SAI children is required to establish normal values of these sensitive indices and enable researchers in this field to perform more effective and timely evaluation, detection, diagnosis, and treatment of different respiratory diseases.

## Competing interests

The authors declare that they have no competing interests.

## Authors' contributions

EGM collected the IOS data from the 26 children, performed the analysis of the IOS data, model parameters, statistical calculations, prepared graphs and tables, and drafted the manuscript. HN conceived, initiated, acquired funding and coordinated all aspects of this research, mentored EGM and helped to write and critically reviewed the different versions of the manuscript. CDM calculated the model parameters for both models by developing improved Matlab codes. MDG mentored EGM and performed the Quality Assurance of the IOS data and elaborated some of the graphs. BD conceived, and directed the initial studies of the eRIC and aRIC models; he also provided the original Matlab codes used for estimating these models' parameters and reviewed the final version of the manuscript. PN contributed to different phases of development of this research and reviewed the final version of the manuscript. All authors read and approved the final manuscript.
